# MITOCHONDRIAL ENERGETICS AND PERCEIVED PHYSICAL FATIGABILITY SEVERITY IN OLDER ADULTS

**DOI:** 10.1093/geroni/igad104.1255

**Published:** 2023-12-21

**Authors:** Emma Gay, Yujia (Susanna) Qiao, Adam Santanasto, Bret Goodpaster, Philip Kramer, Russell Hepple, Steven Cummings, Nancy Glynn

**Affiliations:** University of Pittsburgh School of Public Health, Pittsburgh, Pennsylvania, United States; University of Pittsburgh, Pittsburgh, Pennsylvania, United States; University of Pittsburgh, Pittsburgh, Pennsylvania, United States; AdventHealth, Orlando, Florida, United States; Wake Forest University School of Medicine, Winston-Salem, North Carolina, United States; University of Florida, Gainesville, Florida, United States; California Pacific Medical Center Research Institute, San Francisco, California, United States; University of Pittsburgh School of Public Health, Pittsburgh, Pennsylvania, United States

## Abstract

It has been hypothesized that lower skeletal muscle energetics may contribute to onset of age-related perceived fatigability. Consistent with this idea, previous research found that maximal ATP production (ATPmax) was associated with higher perceived exertion (i.e., fatigability) measured in older adults with the Borg Scale at the end of a slow walking task. We extend this work to SOMMA examining skeletal muscle energetics (ATPmax and maximal complex I&II supported oxidative phosphorylation [maxOXPHOS]) with perceived physical fatigability using the Pittsburgh Fatigability Scale (PFS, range 0-50, higher=greater). PFS captures fatigue perception anchored to activities of varying intensity and duration. We assessed ATPmax in vivo by 31P magnetic resonance spectroscopy following an acute knee extensor exercise bout. maxOXPHOS was quantified ex vivo in permeabilized fiber bundles by high-resolution spectroscopy. Participants (N=721 with PFS and ≥1 energetics measure) were aged 76.4±5.0 years, 56.7% women, 86.7% White. Lower ATPmax (r=-0.21) and maxOXPHOS (r=-0.29) correlated with higher PFS scores (both p<0.001). Linear regressions adjusted for age, sex, technician, and total daily accelerometry-derived physical activity revealed that each one SD lower ATPmax (0.15 mM/sec) was associated with 1.15 (95% CI: 0.50, 1.79) higher PFS scores; each one SD lower maxOXPHOS (18.5 pmol/(sec*mg)) was associated with 1.79 (95% CI: 1.14, 2.45) higher PFS scores. Impaired skeletal muscle energetics may be implicated in the etiologic pathway of age-related fatigability. Next steps will examine the longitudinal association between changes in skeletal muscle energetics and age-related fatigability.

